# Investigating the existence of an osmotic barrier between xylem fibers and vessels in sugar maple (*Acer saccharum*) using microCT

**DOI:** 10.1093/treephys/tpae134

**Published:** 2024-10-17

**Authors:** James A Robinson, Matt Rennie, Mike J Clearwater, Daniel J Holland, Abby van den Berg, Matthew J Watson

**Affiliations:** Biomolecular Interaction Centre & Chemical and Process Engineering Department, University of Canterbury, 20 Kirkwood Avenue, Ilam, Christchurch, 8041, New Zealand; Biomolecular Interaction Centre & Chemical and Process Engineering Department, University of Canterbury, 20 Kirkwood Avenue, Ilam, Christchurch, 8041, New Zealand; School of Science, University of Waikato, Hillcrest Road, Hillcrest, Hamilton, 3216, New Zealand; Biomolecular Interaction Centre & Chemical and Process Engineering Department, University of Canterbury, 20 Kirkwood Avenue, Ilam, Christchurch, 8041, New Zealand; Proctor Maple Research Center, University of Vermont, 364 Marvin Taylor Road, Underhill, VT 05489, USA; Biomolecular Interaction Centre & Chemical and Process Engineering Department, University of Canterbury, 20 Kirkwood Avenue, Ilam, Christchurch, 8041, New Zealand

**Keywords:** sucrose, synchrotron, xylem anatomy, xylem embolism, xylem sap

## Abstract

Sugar maples (*Acer saccharum* Marshall) develop elevated stem pressures in springtime through the compression and expansion of gas bubbles present within xylem fibers. The stability of this gas within the fibers is hypothesized to be due to the elevated sugar concentration of maple sap and the presence of an osmotic barrier between fibers and vessels. Without this osmotic barrier, gas bubbles are predicted to dissolve rapidly. In this work, we investigated the existence of this osmotic barrier. We quantified the fraction of the xylem occupied by gas-filled fibers using synchrotron-based microCT. After imaging fresh stem segments, we perfused them with either a 2% sucrose solution or water, imaging again following perfusion. In this way we directly observed how total gas present in the fibers changed when an osmotic pressure difference should be present, with the 2% sucrose solution, and when it is absent, with the water. Following a first round of perfusion, we perfused stem segments with the other perfusate, repeating this multiple times to observe how switching perfusates affected gas-filled fibers. We found that perfusing stem segments with water resulted in a significant reduction in the xylem fiber gas, but perfusing stem segments with a sucrose solution did not significantly reduce the gas in the fibers. These results support the hypothesis that an osmotic barrier exists between fibers and vessels.

## Introduction

Sugar maple (*Acer saccharum* Marshall) is known to generate elevated stem pressures in response to repeated cycles of freezing and thawing (Jones et al. 1903). The ability of sugar maples to develop elevated stem pressures is believed to be due to processes occurring in the microstructure of the xylem (Milburn and O'Malley 1984, Tyree 1995). Here, we utilize the capacity of high-resolution micro-computed tomography (microCT) to study this microstructure in maple saplings.

Sugar maples have long been known to display elevated springtime pressures (Jones et al. 1903). These pressures develop when maples (in their native climates) regularly undergo periods of slow freezing and thawing, as temperatures drop and rise over a day, with high pressures of over 100 kPa gauge developing after a tree has thawed.

**Figure 1:**
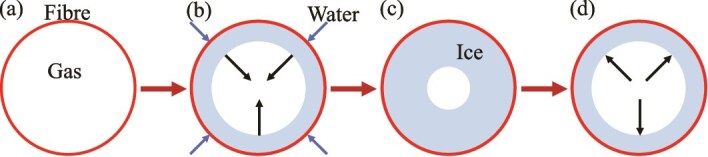
Diagram of the freeze–thaw mechanism showing how pressure develops in an individual xylem fiber. (a) Panel shows the initial condition with the fiber being full of gas. (b) Panel shows the fiber as the tree begins to freeze. As the temperature decreases the gas pressure drops and the pressure differential between the cold gas in the fibers and the water in the vessels draws water from the xylem vessels into the fibers where it freezes to form ice (c). The movement of water into fibers and the volumetric expansion associated with ice formation serves to compress the gas within the fibers. (d) Panel shows the thawing state. As the tree warms, the ice will melt and the gas will increase in pressure, leading to an overall elevated stem pressure. After sufficient time the fiber will return to state (a).

In Milburn and O'Malley (1984), a mechanism for pressure development in sugar maple was proposed (Fig. 1), based on experiments on stem segments. That work proposed that as trees freeze, gas bubbles present in the fibers (fiber embolisms) are compressed as water is drawn into the fibers. During subsequent thaw, the gas heats up, increasing in pressure, leading to an increase in overall stem pressure.

Subsequent authors have contributed improvements to this basic theory. Most recently mathematical modelling has shown that by incorporating water uptake through the roots it is possible to replicate the observed pressure data, including an increase in maximum stem pressure across multiple cycles of freezing and thawing (Ceseri and Stockie 2013, Graf et al. 2015, Zarrinderakht et al. 2024).

**Figure 2:**
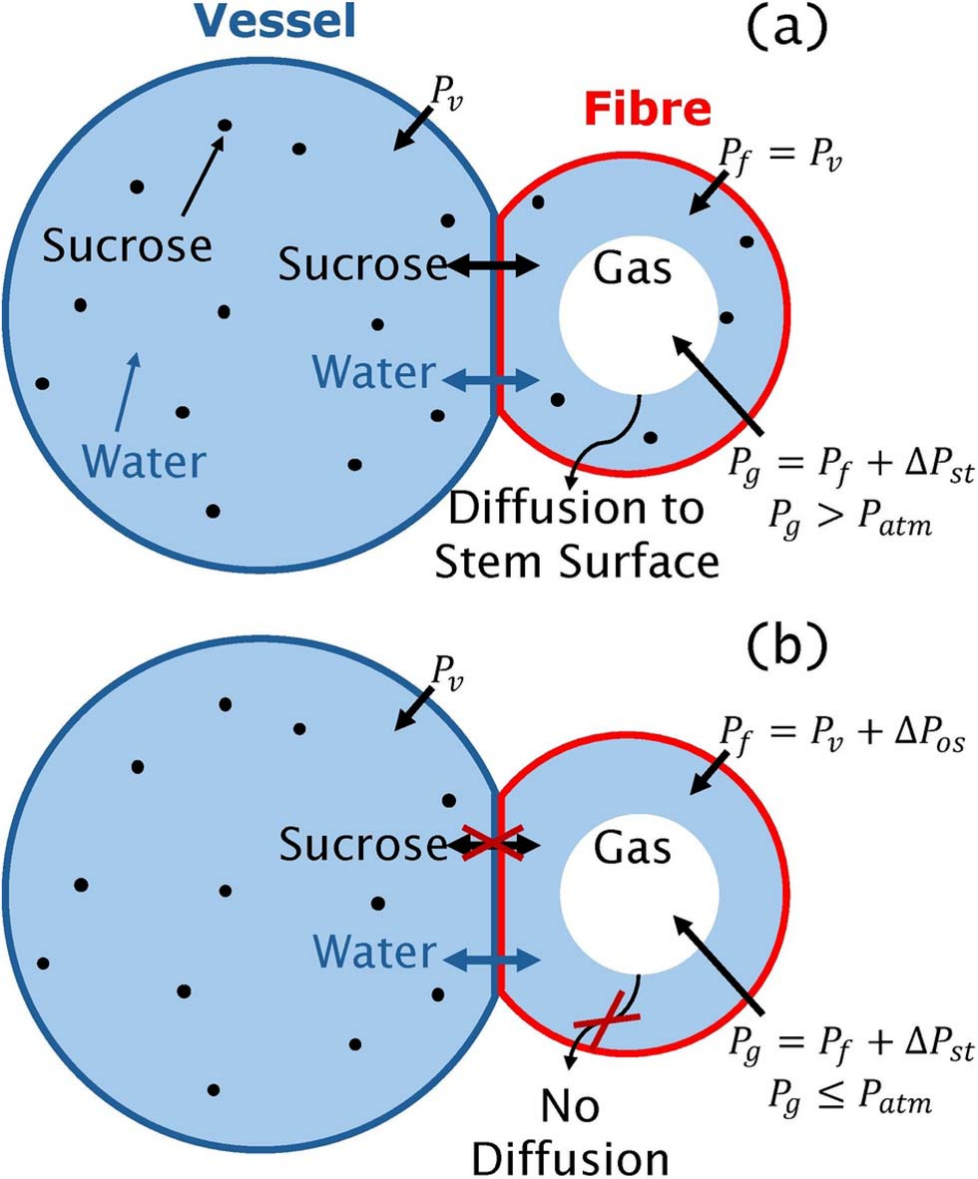
Diagram of the effects of the hypothesized osmotic barrier. (a) Panel shows the case where no osmotic barrier is present and water and sucrose can move freely between fibers and vessels (traversing the cell walls). In this case the liquid pressure in the fibers (${P}_f$) equals the sap pressure in the vessels (${P}_v$). The pressure of the gas bubble in the fiber (${P}_g$) equals ${P}_f$ plus the surface tension pressure difference ($\Delta{P}_{st}$). Under these circumstances, we expect ${P}_g>{P}_{atm}$ where ${P}_{atm}$ is the atmospheric pressure at the tree surface, leading to gas dissolving into solution and diffusing towards the tree surface. (b) Panel shows the case where water can penetrate the fibers but sucrose cannot cross the cell wall. ${P}_f={P}_v+\Delta{P}_{os}$ where $\Delta{P}_{os}$ is the osmotic pressure difference (which is negative) due to the difference in sucrose concentration. The result is ${P}_g<{P}_{atm}$ and no diffusion occurs. While the diagram depicts the fiber as directly in contact with the vessels, there may be other fibers, as well as fiber tracheids, between a fiber and a vessel.

The original theory for maple pressure development relies on the presence of gas in the fibers. If the stem pressure in the vessels is high (equal or greater than the air pressure), as occurs during the thawing phase, then the pressure of the gas within the fibers will be elevated above the air pressure at the stem surface. The high pressure of the fiber gas bubbles will lead to more gas dissolving into the liquid phase, and the gas in the fibers should dissolve over time (Tyree 1995) (see Fig. 2a). The concentration of a species on either side of a gas–liquid interface is dependent on the gas pressure, as dictated by Henry’s law. The presence of any water inside the fibers will elevate the pressures of the gas bubbles due to the effects of surface tension on small bubbles. There is then diffusion of dissolved gas towards the surface of the stem, where liquid is in equilibrium with gas at atmospheric pressure, leading to the gas bubble slowly dissolving over several hours. Therefore, we should not expect gas in the fibers after elevated stem pressures are observed, and the original theory for pressure development was incomplete.

It was proposed that this effect is mitigated by an osmotic barrier in the cell walls between xylem vessels and fibers (Tyree 1995) (see Fig. 2b). This osmotic barrier would prevent sucrose from diffusing into fibers. Maple sap has a very high sugar content (2–4% (Taylor 1956)), which is mostly sucrose. This high sucrose content develops over the winter months into spring (Johnson and Tyree 1992). The presence of sucrose at high concentrations in the vessels, and its absence in the fibers results in an osmotic pressure difference, which depresses the pressure within the fibers. This keeps the pressure low enough that the bubbles do not dissolve and so allows the stem pressure to develop as outlined. It also depresses the freezing point in the vessels, ensuring ice formation begins inside the fibers, which modelling work suggests is key to developing large elevated pressures (Graf et al. 2015).

The significance of sucrose in pressure development was already established experimentally in Johnson et al. (1987). In that work, stem segments were put through controlled freeze–thaw cycles measuring sap uptake and exudation, confirming that fresh stem segments exuded sap upon thaw (consistent with Milburn and O’Malley’s theory). The stem segments were then perfused for 2 h with water, and then a 2% sucrose solution, repeating the freeze–thaw measurements after each perfusion. When perfused with water, stem segments did not exude upon thaw; however, when subsequently perfused with a sucrose solution, the stem segments would again exude upon thaw. This suggests a reversible change occurred in the fiber embolisms.

Later work showed that stem segments perfused by a high molecular weight fluorescent dye did not show any significant penetration into the fibers, consistent with an osmotic barrier being present (Cirelli et al. 2008). However, given the different functional groups present, it is unclear whether sucrose and the fluorescent dye are truly comparable molecules. Cirelli et al. (2008) also showed that the water uptake of stem segments when infused with water or sucrose solution is consistent with an osmotic barrier being present. That work also speculated on the underlying mechanism that would allow for an osmotic barrier. Based on SEM results, Cirelli et al. (2008) suggested libriform fibers lack pits connecting them to vessels. The cell wall thus acts as a semipermeable barrier, which allows water through, but not larger molecules such as sucrose. This hypothesis is supported by the observation that large-molecule fluorescent dyes do not penetrate fibers.

The cell walls of secondary xylem conduits and fibers are known to be porous, containing micropores, which can facilitate the movement of water and other molecules between neighboring cell lumens (Donaldson et al. 2018). These micropores are expected to be on the nanometer scale; Carpita et al. (1979) suggested the micropores are 3–5 nm in other species. We are unaware of any examination of micropores in sugar maple, but the micropores observed in other species are only slightly larger than sucrose (which is approximately 1 nm (Price et al. 2016)). Thus, it is possible that the micropores do not permit diffusion of molecules of the size of sucrose, or that the properties of sucrose may affect its diffusivity. This is all supported by the fact that, perfusing stem segments with other large-molecule sugars (maltose, lactose and raffinose) can produce equivalent behaviour to perfusing with sucrose (Johnson et al. 1987).

Previous studies have provided promising evidence that the presence of sucrose within the xylem vessels contributes to the development of stem pressure in maple (Johnson et al. 1987, Cirelli et al. 2008). However, the involvement of gas bubbles within the fibers, and the proposed stabilizing effect of sucrose on these bubbles, is more difficult to confirm using stem perfusion alone. Over the past decade, microCT has emerged as a powerful tool for non-invasively studying the internal structure of plants in three dimensions (3D) (Karahara et al. 2015). Synchrotron-based microCT is particularly powerful and has been used to study the formation and repair of xylem vessel embolisms in other tree species (Brodersen et al. 2013, Brodersen et al. 2018, Lintunen et al. 2022). The approach has been used to image vessel embolisms in sugar maple (Driller et al. 2020, Robinson et al. 2023). However, prior studies on maple have lacked the resolution needed to resolve fiber embolisms. Studies on other species done at much higher resolutions only focused on xylem vessel embolisms (Brodersen et al. 2018, Secchi et al. 2021), while fiber embolisms are key to the pressure development mechanism in sugar maple.

The aim of this was work was to examine the existence of the hypothesized osmotic barrier between fibers and vessels by looking at how fiber embolisms changed when stem segments were perfused with different solutions and whether those changes were consistent with theory. We used high-resolution synchrotron-based microCT to image fiber embolisms within freshly cut stem segments, and then in those same stem segments following successive rounds of perfusion with water or sucrose solution. Alongside using microCT to image fiber embolisms, we also examined how the weight of the stem segments changes after each perfusion round to monitor water uptake or loss.

## Materials and methods

### Plant material

One to -two year-old sugar maple (*A. saccharum* Marshall) saplings were purchased from a nursery in Melbourne, Australia at the start of the experiments (April 2023, autumn in the Southern hemisphere). These saplings were 70–90 cm tall with a root collar diameter of 7–20 mm. They each had two to three large branches. The saplings leaves were almost all orange, suggesting they were entering dormancy.

The experiments took place over 2 days, with samples cut from each sapling shortly before their initial imaging, a state subsequently termed fresh. Eight fresh samples were taken on the first day and an additional four were taken on the second. Samples were excised with secateurs from the either the main stem or one of the larger branches, with samples taken where the diameter was narrow, but the stem surface was not green. By cutting from branches, multiple samples could be taken from each saplings (up to 3).

The cut stem segments were ~2.5–3.5 mm in diameter. The small diameter was necessary to ensure the samples fitted within the narrow field of view of the beamline. The samples were ~5–8 cm long. The length was kept long enough to allow easy mounting to the perfusion rig, whilst being short enough that when mounted in front of the beamline, where they were held in place at one end only, they would be rigid enough to prevent movement artefacts while imaging.

The samples were cut while in air, which has been known to induce embolisms in vessels (Wheeler et al. 2013). Although cutting in air can induce embolisms in vessels, we are concerned with changes in fiber embolisms after perfusion. Fibers are shorter (~400 μm (Driller et al. 2023)) isolated cells. We imaged samples >10 mm away from either end, thus minimizing the effect of any severed fibers.

### Perfusion

**Figure 3:**
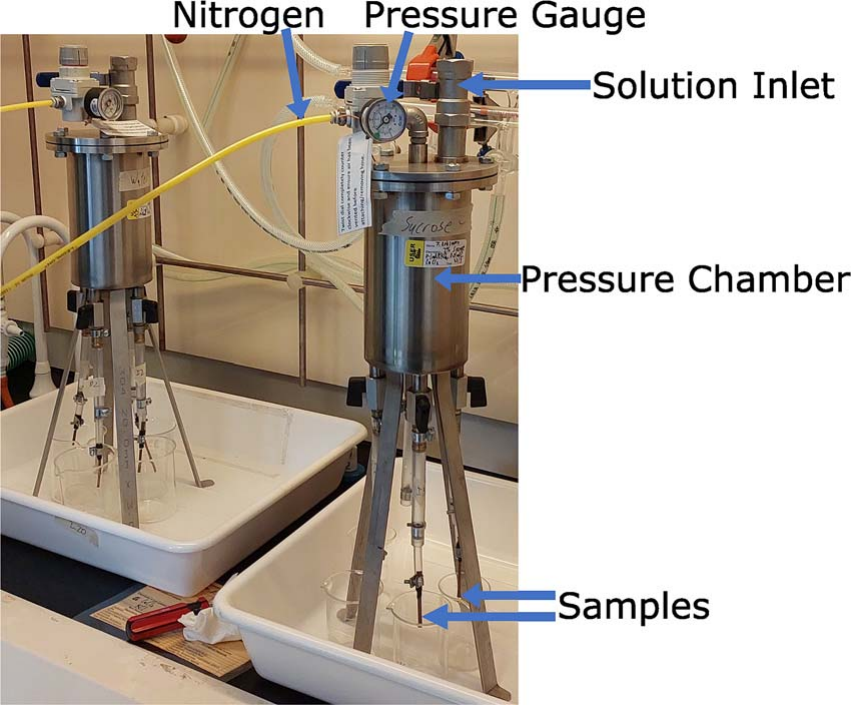
Perfusion rigs used to perfuse samples with sucrose solution (front) and water (back). The two rigs are identical in construction.

Stem segments were perfused using a custom experimental rig (Fig. 3). This apparatus enabled us to perfuse four stem segments simultaneously with a pressurized perfusate. We ran the perfusion at 50–60 kPa gauge, via a pressure regulator connected to a compressed nitrogen cylinder. We utilized two separate perfusates: a solution of 0.001 mol L^−1^ CaCl_2_, 0.01 mol L^−1^ KCl (referred to as water) and a solution of 0.001 mol L^−1^ CaCl_2_, 0.01 mol L^−1^ KCl and 2% sucrose (referred to as sucrose solution). These solutions were prepared with deionized water. The salts added are typical for perfusates (Torres-Ruiz et al. 2017).

The concentration of the sucrose solution, the perfusion pressure and the perfusion time were deliberately chosen to be consistent with that of Johnson et al. (1987), who first showed the necessity of sucrose in sap through similar perfusion experiments (though with samples with much larger diameter and length). The perfusion pressure was lower than the high pressures sometimes used to flush embolisms from the vessels of excised stem segments (Sperry et al. 1988) and should not be sufficient to flush embolisms from closed fibers. While perfusion under positive pressure may have added to the gas pressure with the fibers, as would occur in vivo during periods of positive stem pressure, the water and sucrose solutions were perfused at the same pressure. The sucrose concentration was within the expected range observed for winter maple sap (Taylor 1956) and below the concentration required to have a significant effect on surface tension and capillary effects (Docoslis et al. 2000). It was therefore assumed that any difference in fiber gas content between the two treatments was caused by the effect of sucrose on the solute potential of the perfusate.

Using this perfusion setup we ran two experiments, with the difference between the two being perfusion time. For the first experiment, we took 12 samples (8 on day 1 and 4 on day 2) and imaged them freshly cut (referred to as fresh). We then took half of the samples (samples S1 through S6) and perfused them with sucrose solution for 2 h. The other half of the samples (samples W1–6) were perfused for 2 h with water. Following a perfusion, we again imaged the samples. The samples were then returned to the perfusion rig to perfuse with the other perfusate. We alternated the perfusion a total of three times. That is, we have samples that were initially perfused with sucrose solution (samples S1–6), then water, then sucrose solution, and samples initially perfused with water (samples W1–6), then sucrose solution, then water.

The perfusion time of 2 h was chosen to match that used in Johnson et al. (1987). Their work showed reversible changes in stem behaviour after 2-h perfusions, suggesting it should be a sufficient length of time to induce changes in fiber embolisms.

Not all samples taken at the start of each day were taken through the full three rounds of perfusion. This is because some samples were broken while being mounted onto the perfusion rig. If both of the pieces were too small to remount for subsequent perfusion then the sample could no longer be used. Additionally, when samples W1–W3 and S3–S5 were undergoing their third perfusion a leak occurred (where one sample was mounted) causing the perfusion solution to be drained and the gas cylinder to empty through the leak. Once noticed and corrected, they were perfused for a further 20–30 min, giving a total perfusion time of between 1 and 2 h. The data are included for completeness.

Flow rates through the perfusion rig were not recorded. Stem segments typically produced a single droplet of perfusate per minute, though this varied significantly between samples. A few millilitres were collected in beakers placed below the samples, but given the length of the perfusion some of this perfusate would have evaporated, and so the volume collected was not representative of the volume perfused. The first eight samples were perfused without orientation being noted. Subsequent samples were all aligned to be perfused from the root to the canopy.

Samples could not be perfused while mounted in the microCT stage and the sections being imaged were small (approximately 3 mm). It was thus not feasible to perfectly align all samples in the time available and so when imaging the individual stem segments after each perfusion the region of the stem imaged was not kept exactly the same.

The initial results obtained from the alternating 2-h perfusion experiments supported that 2-h was sufficient to see differences in results following water vs sucrose perfusion (Fig. 5b and e). However, we also wanted to determine if the results following sucrose perfusion remained consistent after perfusion for extended periods of time. As such, two of the samples initially perfused with sucrose (S1 and S2) were set up alongside an additional two fresh samples (S7 and S8) and perfused for an extended period with sucrose solution. The intended perfusion time was >8 h; however, the equipment again developed a leak and lost pressure. This was corrected when noticed leading to an estimated perfusion time of >3 h, still considerably longer than the 2-h perfusions.

Tables S2 and S3 available as Supplementary data at *Tree Physiology* Online summarize the perfusion treatments for each sample.

### Weight measurements

In total, there were 14 samples. 13 of the samples were weighed using a laboratory balance (Mettler Toledo AB104-S) when freshly cut and all 14 were weighed after each round of perfusion. At the end of the perfusions, 13 samples were dehydrated in an oven at 80 °C for 24 h and their dry weight recorded. Seven samples were dried for a further 24 h and showed no significant additional mass loss. Water content was calculated from these measurements as $WC=\left( MAS{S}_{wet\ weight}- MAS{S}_{dry\ weight}\right)/ MAS{S}_{wet\ weight}$, the mass of water in the sample (the wet weight minus the dry weight) divided by the sample’s wet weight.

We also calculated the relative change in water content from fresh by taking the current water content (after a perfusion round), subtracting the fresh water content, and then dividing the resulting value by the fresh water content to get the relative change $\left(\left(W{C}_{current}-W{C}_{fresh}\right)/W{C}_{fresh}\right)$.

### Synchrotron-based microCT

The microCT beamline (known as the MCT beamline) at the ANSTO Australian synchrotron in Melbourne Australia was used for imaging experiments. The beamline was run using a mono-beam at 18 keV. The energy level was chosen after preliminary scans showed this value appeared to produce images with good contrast between embolized and non-embolized features. MicroCT works by taking a series of projection X-rays at different angles and from these reconstructing a 3D image. For our imaging, we took 1800 projections each 0.1° apart, spanning a rotation of 180°. The imaging exposure time was 0.06 s and taking a full scan took ~10 min due to the rotation speed of the stage. It took 15–45 min from a sample being removed from the perfusion rig to being imaged, during which time samples were laid horizontally and their ends covered with parafilm. There was another 15–45 min following imaging before samples were remounted for perfusion with their next perfusate.

**Figure 4:**
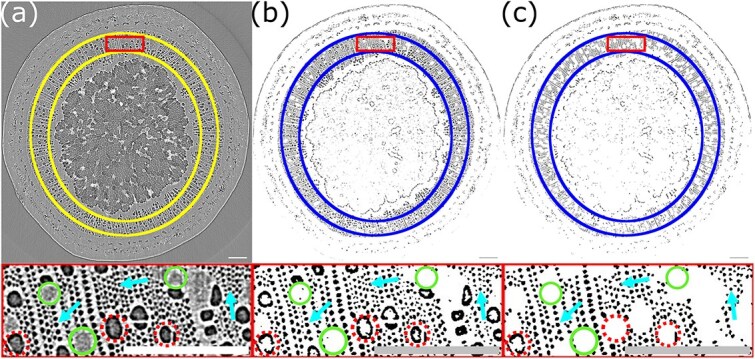
Example MicroCT reconstructed image of a transverse section of a freshly cut maple stem. (a) Panel shows the image, (b) panel shows the image after thresholding and (c) panel shows the image after additional postprocessing to remove vessel embolisms. The yellow rings (a) and blue rings (b and c) denote the region selected as the xylem, for analyzing the fraction of the xylem embolized. A close-up view of the region within the box is shown below each image. Dotted circles show examples of embolized vessels and solid circles show sap filled vessels. Arrows point to examples of groups of fibers. All visible fibers appear to be embolized. The scalebar in these images is 0.25 mm.

The 1800 X-ray image projections were reconstructed using custom software provided by the beamline on the ANSTO’s ASCI computing system. The reconstruction produced 3D images of a small region of the stem segment, 3.46 mm long. The total image resolution was 2560 × 2560 × 2160 voxels with an effective voxel resolution of 1.6 μm^3^. The first 250 and last 260 slices of this image (slices refers to a layer of the 3D image at a fixed vertical position) were discarded as these images had a much smaller signal range than the rest of the stack, which would negatively impact any thresholding. The final images (see Fig. S3 available as Supplementary data at *Tree Physiology* Online and Fig. 4a) analyzed were thus 2560 × 2560 × 1650 in size, corresponding to a 2.64-mm long section of the stem.

### Analysis approach

Considering individual slices of the 3D images produced (see Fig. 4a), embolized structures in the xylem, either fibers or vessels, were visibly identifiable due to the lower signal in these regions resulting in a darker appearance than surrounding xylem tissue and/or water-filled cells. Phase contrast due to the sharp change in density also results in a higher intensity border (appearing white) around embolized structures. Here, the analysis focuses on the fiber embolisms, as these are important for springtime pressure development in maples. However, we note that some analysis of the vessel embolisms was performed (see Supplementary data).

Postprocessing of images was done using the Fiji distribution of imagej (Schindelin et al. 2012). Each 3D image was read into imagej as a stack of 1650 slices. These slices were then processed through a series of steps, involving thresholding to isolate embolized fibers and vessels (Fig. 4b), and then subsequently remove most vessel embolisms (Fig. 4c). Both libriform fibers and fiber tracheids are present in sugar maple, with prior work suggesting that libriform fibers play the main role in springtime pressure development (Cirelli et al. 2008). Distinguishing between fiber types is challenging, even under high-resolution electron microscopy (Driller et al. 2023), and was not feasible with our data.

Further detail on the processing done to remove vessel embolisms is given in the Supplementary data, and the full postprocessing algorithm is included as a supplemental file.

The 3D stack of processed slices (1650 in total) was read into Matlab (The MathWorks Inc. 2022). We manually defined a region of the xylem to analyze across all slices (the two yellow circles in Fig. 4a). For each slice, the number of embolized pixels (shown in black in Fig. 4c) in the defined region of analysis was calculated alongside the total no. of pixels in this region. Because we have removed almost all vessel embolisms this embolized area represents the area of the xylem occupied by fiber embolisms. We then summed the no. of embolized pixels (the embolized area) across each slice and divided by the sum of the total no. of pixels in the defined region (the xylem area) across each slice. This gave us the fraction of the xylem occupied by embolized fibers averaged across all slices analyzed (referred to hereafter as the embolized fiber fraction). We also compute an experimental uncertainty for the embolized fiber fraction. This is done by calculating the embolized fiber fraction for each individual slice (the no. of embolized pixels divided by the total no. of pixels in the xylem region for that slice), applying a rolling average to smooth out any fluctuations associated with noise, and then taking the maximum and minimum of this smoothed distribution. This is further detailed in the Supplementary data.

Alongside the few remaining vessel embolisms, there were also some additional sources of error arising from signal variability across the stack and the effects of minor motion of the stem, which can result in slight blurring in regions of the image. All of these factors impacted how well fibers threshold in different regions of the 3D image. However, the error introduced by such issues should be similar for all images taken, and we focused on comparative analysis between fresh and perfused samples rather than quantitative analysis of a single stem. Therefore, the influence of these errors on the analysis is likely to be minor.

Once the embolized fiber fraction was calculated, we conducted statistical analysis on the data. We utilized a series of one-tailed paired *t*-tests to compare the embolized fiber fraction before and after each perfusion (done separately for each sample). This allowed us to evaluate whether there was a statistically significant decrease in the embolized fiber fraction after each perfusion round. This analysis is further detailed in the Supplementary data.

**Figure 5:**
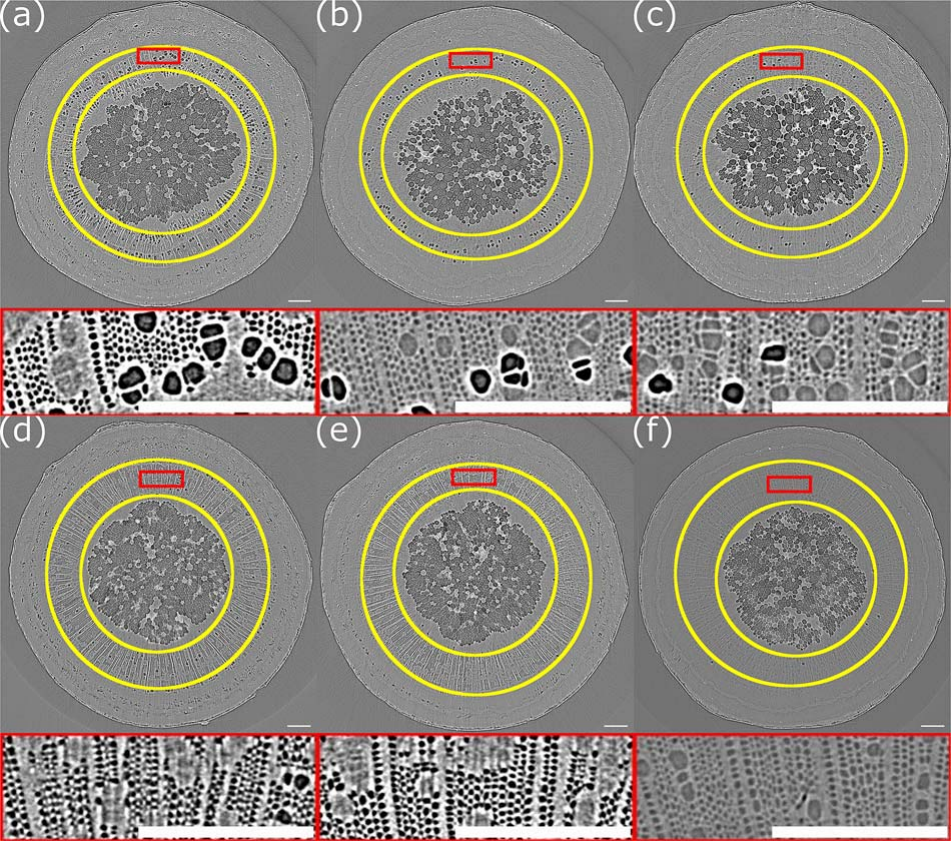
MicroCT images taken of stem sections. (a–c) Panels show a stem segment, which is initially perfused with water. (a) Is that stem segment fresh, (b) is after a 2-h water perfusion and (c) after a 2-h sucrose solution perfusion. (d–f) Panels show a stem segment initially perfused with sucrose solution. (d) Is that stem segment fresh, (e) after a 2-h sucrose perfusion and (c) after a subsequent 2-h water perfusion. The images are not of the exact same region of each stem section as when imaging samples in the MicroCT following a perfusion there was no simple way to ensure the same region of the stem was being imaged or that the stem orientation was the same (azimuthally) as prior to the perfusion. Below each image is a close up of the region highlighted by the box. The scalebar in all images is 0.25 mm in length.

## Results

The resolution of the microCT was sufficient to visually resolve the xylem elements of interest (fibers and vessels) (Fig. 5). Gas-filled (embolized) cells have a much lower signal intensity and appeared dark on the images. The maple stems displayed heavy initial fiber embolization (Fig. 5a and d). The extent of the initial embolization varied between samples, but for all fresh samples the majority of the fibers appeared embolized. Following each perfusion treatment, changes in embolized fibers were observed (Fig. 5b, c, e, f). These changes were characterized across all samples by looking at the embolized fiber fraction, calculated as outlined in the Materials and methods section.

### Samples first perfused with water

**Figure 6:**
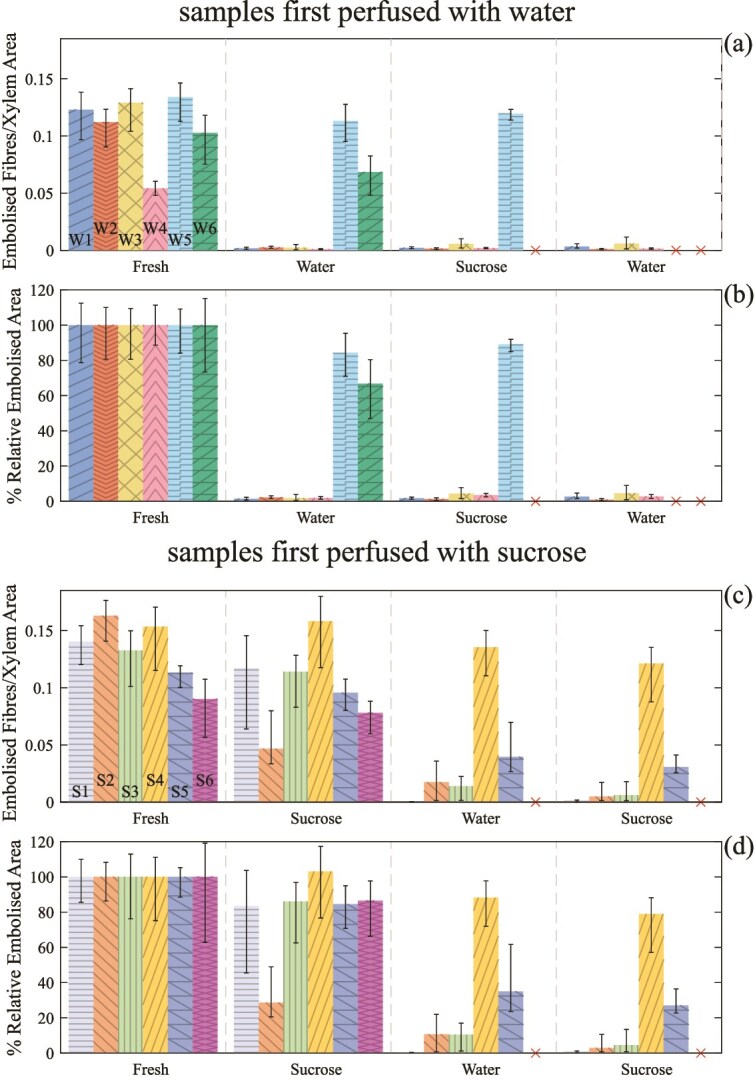
Fraction of xylem occupied by embolized fibers in each sample when fresh, and after each perfusion with different perfusates. Panels (a) and (b), respectively, show the measured embolized fiber fraction and the embolized fiber fraction relative to the initial embolized fiber fraction, both for samples initially perfused with water (samples W1–6). Panels (c) and (d), respectively, show the measured embolized fiber fraction and the embolized fiber fraction relative to the initial embolized fiber fraction, both for samples initially perfused with sucrose solution (samples S1–S6). Results are ordered from those that went through the most rounds to those that went through the fewest. A cross through the *x* axis indicates perfusion was not done for that sample. The error bars in (a) and (c) are the max and min embolized fiber fraction across each slice (1650 in total) of an image, after a rolling average was applied to reduce noise (see Supplementary data for further detail). For the relative values, (b) and (d), the error bars are the same, just divided by the fresh embolized fiber fraction.

The embolized fiber fraction for samples first perfused with water was initially around 0.1–0.15 (with only one sample being below 0.1) (Fig. 6a). Following the perfusion with water, four out of the six samples experienced a large decrease in embolized xylem area to ⪅10% of their fresh embolized fiber fraction (Fig. 6b). The other two samples (W5 and W6) also showed a decrease in embolized area, though the change was less pronounced. For sample W5, the change was small enough that it could be attributable to error. The shift for all samples were evaluated using a paired *t*-test. The *P*-value comparing the embolized fiber fractions before and after the first perfusion was 0.0049. This *P*-value shows that the embolized fiber fraction after the first perfusion is significantly lower, at the 95% level (see Table S1 available as Supplementary data at *Tree Physiology* Online). For the five samples taken through a second perfusion round, there were small shifts, but no large changes in embolized fiber fraction (Fig. 6a and b). Any changes following the third perfusion round were also negligible.

**Figure 7:**
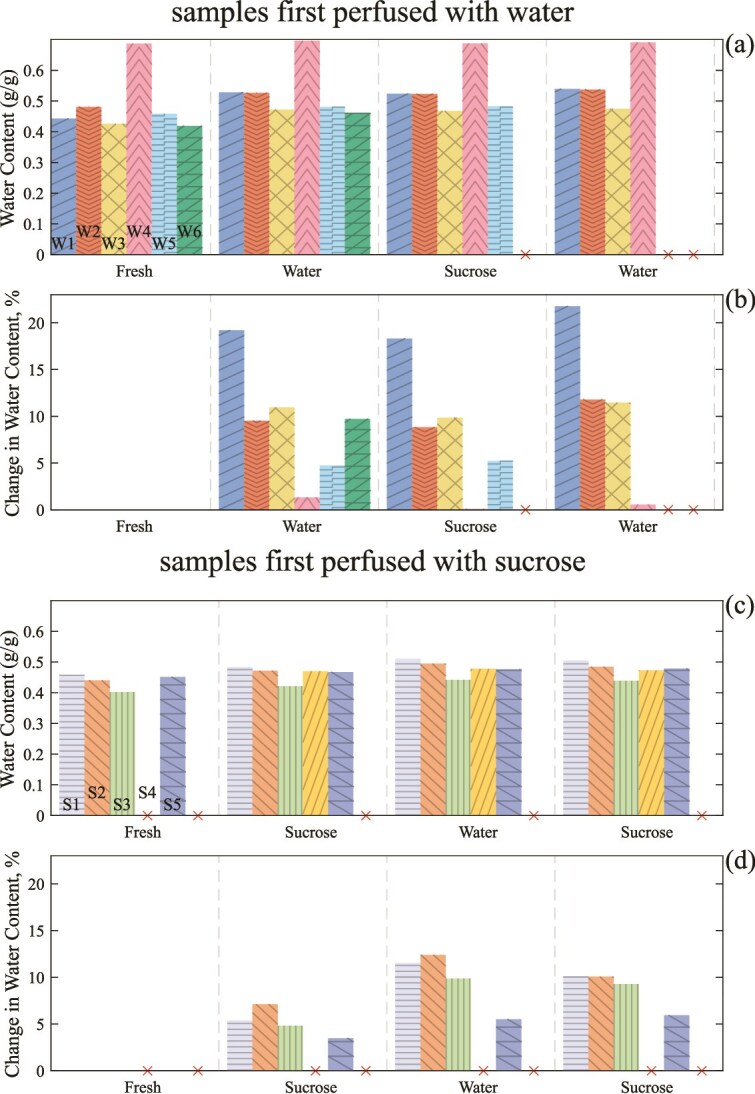
(a) Panel shows the water content of samples fresh and following each round of perfusion, for the samples initially perfused with water, and panel (b) shows the change in water content for those samples from fresh relative to the initial water content, with both being for the samples initially perfused with water. (c) Panel shows the water content of samples fresh and following each round of perfusion, for the samples initially perfused with sucrose solution, and panel (d) shows the change in water content for those samples from fresh relative to the initial water content, with both being for the samples initially perfused with water. Colors/patterns are as in Fig. 6. A cross through the *x* axis indicates where there is no data for that sample.

The water content (Fig. 7a) of the fresh samples was typically 0.4–0.5 g g^–1^, with a single sample from the set having a high water content at 0.68 g g^–1^ (W4 in Fig. 7a). To highlight the changes following each perfusion, we again plot a relative measure (Fig. 7b).

Following perfusion with water all samples, even those that did not show significant changes in xylem embolization, displayed an increase in water content of 5%–10%. One sample (W1) displayed a much higher water uptake, at 20%. This sample had a high fresh embolized xylem fraction (~0.1) and a very high embolized vessel area (see Fig. S2 available as Supplementary data at *Tree Physiology* Online), which likely contributes to the high water uptake. Following perfusion with sucrose solution (the second perfusion), four out of the five samples had a slight decrease in water content. Following another perfusion with water (the third perfusion overall), a further increase in water content was observed. Two samples, W5 and W4, did show much lower water uptake across this series.

### Samples first perfused with sucrose solution

For five out of six samples first perfused with sucrose solution there was a reduction in the embolized fiber fraction following perfusion with sucrose solution (Fig. 6). However, for four of those five (S1, S3, S5 and S6) this reduction was small, decreasing to 80% of their initial embolized fiber fraction (Fig. 6d). One sample (S2) had a larger reduction to 20% of the initial embolized fiber fraction. One sample (S4) instead showed a slight increase. Comparing the results of the first perfusion using a paired t-test gave a *P*-value of 0.072, which falls outside the 95% confidence level (though is still within the 90% level) meaning there is insufficient evidence to confirm that the embolized area has decreased (see Table S1 available as Supplementary data at *Tree Physiology* Online).

For the five samples taken through the second perfusion round (perfused with water), all samples showed a reduction in fiber embolization. Two samples showed a small reduction (S2 and S4) following the perfusion. The other three showed a decrease in the embolized fiber area of more than 0.05 (and more than 0.1 for two of the three) from the values after the first perfusion. After the third perfusion (with sucrose solution), there was minimal change in fiber embolization.

Water content was measured for samples S1–S5 (Fig. 7c and d). Water content for sample S4 was calculated only after perfusion as the fresh weight was not recorded. Relative water content values for sample S4 were not calculated for this reason (Fig. 7d).

Upon initial perfusion with sucrose all samples showed an increase in water content ranging from 7% of the fresh water content (S2) to 4% at the lowest (S5) (Fig. 7c and d). A further increase in water content occurred upon subsequent perfusion with water. Subsequent perfusion with sucrose appeared to lead to a small decrease in water content.

### Extended perfusion samples

**Figure 8:**
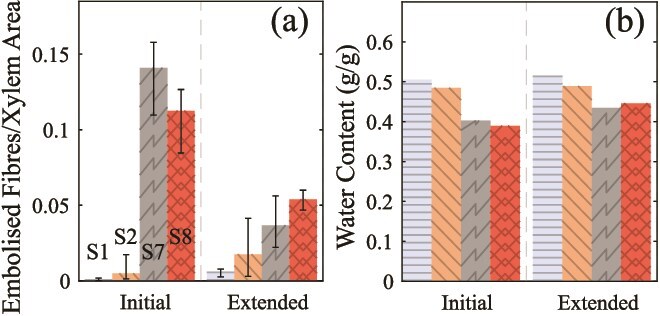
Results of the extended perfusion, before (initial) and after the extended perfusion. (a) Panel shows the measured embolized fiber fraction. (b) Panel shows the water content of samples. For the four samples (S1, S2, S7, S8), S1 and S2 were previously used for the 2-h perfusion experiments, so their initial condition is after a third 2-h perfusion round with sucrose solution. S7 and S8 were taken through the extended perfusion from fresh (their initial state). The error bars in (a) are the max and min embolized fiber fraction across each slice of an image, after a rolling average was applied to reduce noise.

For the two samples taken through three perfusions and then an extended perfusion with sucrose solution (samples S1 and S2), we saw negligible change in fiber embolization after the extended perfusion (Fig. 8a) or in their water content (Fig. 8b). Extended perfusion with sucrose for samples S7 and S8 (perfused from fresh) showed a larger reduction in the embolized fiber fraction (to 25% for S7 and 48% for S8) than samples perfused for only 2 h with sucrose solution. However, with only two samples, it is difficult to make any definitive statement as to the significance of this reduction. Qualitatively, we note the extent of this reduction was not as large as seen in most samples following a water perfusion, where the embolized fiber fraction decreased to <10% of its initial value, or less than 0.02. The two samples both had high water uptake, though S8 clearly exceeded S7 (Fig. 8b).

## Discussion

The necessity of sucrose content on springtime pressure development has been established by the experimental work in (Johnson et al. 1987). The hypothesized role sucrose plays in pressure development is to keep fiber embolisms stable over time (Tyree 1995). It has been proposed that an osmotic barrier exists between fibers and vessels. The resulting osmotic pressure difference keeps fiber embolisms from dissolving. Fiber embolisms are crucial in developing elevated stem pressures during thaw. Therefore, the osmotic barrier is also important for producing elevated stem pressures. The existence of an osmotic barrier is supported by the infusion experiments in Cirelli et al. (2008), showing water uptake and pressure curves consistent with an osmotic barrier when stem segments are infused with water versus sucrose solution.

Our approach allows us to directly observe fiber embolisms. If an osmotic barrier is present then, when perfusing stem segments with sucrose solution, the osmotic pressure difference should be maintained, and we expect no change in fiber embolisms. Perfusing stem segments with water will remove the sucrose present in the vessels and should eliminate the osmotic pressure difference, resulting in fiber embolisms dissolving.

The results measured from samples following the first round of perfusion provide compelling evidence for the existence of an osmotic barrier between vessels and fibers. For the samples initially perfused with water, four out of six showed a large decrease in embolized fiber fraction following perfusion with water. This change could be visually seen in microCT images (Fig. 5), where fresh samples (Fig. 5a) displayed significant fiber embolization and following perfusion almost all fibers appeared filled (Fig. 5b). For the samples initially perfused with sucrose solution, after the first perfusion, there was only a small decrease in embolized fiber fraction. Additionally, after a subsequent perfusion with water, four out of five samples showed a significantly larger decrease in embolized fiber fraction.

There were some outliers that deviate from this typical response, most notably samples W5 and S4. Sample W5 did have a large amount of its xylem occupied by embolized vessels (see Fig. S2 available as Supplementary data at *Tree Physiology* Online), and sample S4 had a relatively small xylem area. It is thus possible these stem segments had a higher initial resistance to flow, preventing complete perfusion and the full effects of the changes between water and sucrose solution on fiber gas content. However, while we did observe variation between stems in the flow of perfusate, the actual flow rates were not recorded.

Considering the typical response across the majority of samples, these results are consistent with the existence of an osmotic barrier. When samples were perfused with sucrose solution, there is an osmotic pressure difference between fibers and vessels, fiber embolisms are kept stable and there is minimal change in the embolized fiber fraction. When samples are perfused with water the osmotic pressure difference is lost and fiber embolisms dissolve, leading the large observed decrease in embolized fiber fraction.

The response observed via microCT also appears consistent with the changes in sample weights. Samples initially perfused with water, which have fibers filling with water as embolisms dissolve, had larger increases in water content after their first perfusion (with water) than did samples initially perfused with sucrose solution. There were two outliers showing minimal uptake (W4 and W5). W4 had a high a high initial water content, and low initial xylem embolization, suggesting less capacity for additional water uptake, while W5 showed little change in xylem embolization after perfusion, consistent with minimal water uptake.

The samples initially perfused with sucrose solution did still have an increase in water content after their first perfusion, but this increase was small. We speculate that this increase is due to water uptake in regions of the stem aside from the xylem fibers (e.g., pith, parenchyma, phloem, etc). Though Fig. S2 available as Supplementary data at *Tree Physiology* Online shows there is not a clear trend of reduced vessel embolisms across all samples, suggesting cavitated vessels are not the main source of water uptake. The samples initially perfused with sucrose solution displayed a further increase in water content after a second round of perfusion (with water), consistent with the filling of fiber embolisms, once the osmotic pressure difference has been removed.

The results discussed provide support for the existence of an osmotic barrier in sugar maple, because the changes in fiber gas content in response to the presence or absence of sucrose matched the responses predicted based on the existence of a barrier. However, even disregarding the noted outliers, not all observed behavior observed is as expected. First, the samples initially perfused with sucrose solution displayed a decrease in embolized fiber fraction following initial perfusion (with sucrose solution). This decrease was not statistically significant (95% level), but it occurred for almost all samples and the two samples solely perfused for an extended period with sucrose solution both showed a larger reduction in embolized fiber fraction. In all cases, this decrease was smaller than typically observed following water perfusion, suggesting the sucrose is acting to slow gas dissolution. Fiber embolisms will dissolve if their pressure exceeds the air pressure at the stem surface (Tyree 1995), which could occur if the combination of surface tension and applied perfusion pressure was too high. For a 2% sucrose solution, the osmotic pressure difference is approximately −160 kPa (at 20 °C from (Slavik 1974)). Our operating pressure was 150–160 kPa absolute, meaning the liquid pressure in the fiber embolisms should be approximately 0 kPa. The pressure inside the fiber embolisms will thus be ~18 kPa due to surface tension (see Supplementary data for calculation). This pressure is still below atmospheric pressure and so is insufficient to cause embolisms to dissolve. Therefore, should an osmotic barrier be present, fiber embolisms should be stable, and there should be no change following perfusion with sucrose solution. More data are needed to be confident that there is a decrease in fiber embolisms with sucrose perfusion, and at this stage, we cannot confidently speculate as to the cause.

The second unexpected response is that samples perfused with sucrose solution, following prior perfusion with water, do not display any increase in embolized fibers. As noted, the significance of sucrose in pressure development was established by perfusion experiments, which showed that sap exudation capacity was lost when samples were perfused with water and regained following perfusion with sucrose solution (Johnson et al. 1987). Given the mechanism proposed in Tyree (1995), a loss of exudation capacity would correspond to a decrease in fiber embolisms, and a restoration of exudation capacity to an increase in fiber embolisms. However, we do not observe any such change in the measured embolized fiber fraction. Additionally, the infusion experiments in Cirelli et al. (2008) showed that when infusing stem segments with water, uptake smoothly increased with pressure. Under those conditions, we would expect fiber embolisms to dissolve. When infusing those same stem segments with sucrose solution, uptake remained at 0 until the applied pressure was sufficiently high, consistent with an osmotic barrier and the continued presence of fiber embolisms. Those results again suggest the effects of water and sucrose perfusion should be reversible, yet we did not observe any reversibility in our experiments.

It is possible that the unexpected result here is not that fiber embolisms do not increase following perfusion with sucrose solution, but instead that embolisms fully dissolved following a 2-h perfusion with water (consistent with what is observed in Fig. 4b and c). The response observed in Johnson et al. (1987) and Cirelli et al. (2008) could occur if fiber embolisms do not have time to fully dissolve, but instead only shrink in size, when samples are perfused with water. The embolisms can thus be fully restored when the samples are perfused with sucrose solution.

In our case, we observed full fiber refilling. As noted, the expected osmotic pressure generated by the hypothesized osmotic barrier is around −160 kPa, resulting in a 0 kPa pressure in the fibers. Vessels can sustain significantly lower negative pressures (<−1 MPa) without cavitating (Venturas et al. 2017). While tiny microbubbles may be present, which cannot be resolved, the effects of surface tension (which increase bubble pressure as its size decreases) could prevent these bubbles from expanding under the low osmotic pressure. Prior work has shown that perfusing high water content maple stems, where the fibers are presumably filled, with sucrose solution does not restore exudation capacity (Johnson and Tyree 1992), again implying that small fiber embolisms are required to reestablish exudation capacity through sucrose perfusion.

If the issue is that we observe complete fiber refilling, then this would suggest the perfusion time was too long, or pressure too high. Both properties were deliberately chosen to be consistent with Johnson et al. (1987). In Cirelli et al. (2008), samples infused with water were allowed to sit for 1 h before sucrose infusion, though at atmospheric pressure. Both works used significantly larger samples, which would affect the dissolution rate. Johnson et al. (1987) also put samples through a freeze–thaw cycle. Freezing drives small gas bubbles out of solution. It is possible that these bubbles expand upon thaw, driven by temperature increase and the osmotic pressure difference, resulting in some recovery of exudation capacity even with complete fiber refilling. Johnson and Tyree (1992) observed some sap exudation from high water content (>0.8) samples when taken though a freezing/thawing cycle, though this sap exudation was reduced compared with low water content samples.

Both Johnson et al. (1987) and Cirelli et al. (2008) utilized extremely different experimental approaches, which makes it difficult to be confident when comparing the data obtained with these prior studies. As such at this sage, we can only speculate as to the causes of the unexpected behavior observed. Despite this, the results from the first perfusion round do support the hypothesis that an osmotic barrier is present between fibers and vessels, consistent with existing experimental work (Johnson et al. 1987, Cirelli et al. 2008).

## Conclusion

In this work, we used microCT imaging to examine changes in fiber embolisms within maple xylem following perfusion with water or sucrose solution. We demonstrated that microCT was a viable approach for examining maple anatomy and water content at high resolution without sectioning, and thus can be used to study the phenomena associated with stem pressure development, including fiber embolism evolution, in stem segments of this species.

Using microCT, we observed that samples perfused with water showed a large reduction in fiber embolisms, whereas those perfused with sucrose solution showed a much smaller reduction in fiber embolisms. These observations support the existence of an osmotic barrier between fibers and vessels.

However, we still observed some loss of fiber embolisms following perfusion with sucrose solution. We also saw that perfusing with sucrose solution following a water perfusion did not lead to a recovery of the fiber embolisms, in contrast to expectations from prior work. While this study provides further support for the existence of an osmotic barrier, our inability to explain these unexpected behaviors suggest the role sucrose plays in pressure development may be more complex than previously theorized. This work is based on a limited dataset, and there is a need for further work exploring a greater range of samples to fully clarify the role of sucrose in pressure development.

## Supplementary Material

Appendix_v5_tpae134

POSTPROCESSING_FULL_tpae134

## Data Availability

The data presented in this article are available from the authors upon reasonable request.
