# Supplementary material for: Investigating the existence of an osmotic barrier between xylem fibers and vessels in sugar maple (Acer saccharum) using microCT
Source: Tree Physiol. 2024 Oct 17;46(13):108–18. doi: 10.1093/treephys/tpae134 (PMC13016655; doi:10.1093/treephys/tpae134)
Supplement: Appendix_v5_tpae134 [file appendix_v5_tpae134.docx]

Supplemental Material for “Investigating the existence of an osmotic barrier between xylem fibres and vessels in sugar maple (*Acer saccharum*) using microCT”

J. A. Robinson^1*^, M. Rennie^1^, M. J. Clearwater^3^, D. J. Holland^1^, A. van den Berg^2^, M. J. Watson^1^

^1^Biomolecular Interaction Centre & Chemical and Process Engineering Department, University of Canterbury, Christchurch, New Zealand

^2^ Proctor Maple Research Center, University of Vermont, Underhill, Vermont, United States

^3^School of Science, University of Waikato, Hamilton, New Zealand

# Image processing prior to analysis

In order to analyse each 3D image, to extract information on the embolised fibres, image processing was done using the Fiji distribution of imagej (Schindelin et al. 2012). Each 3D image was read into imagej as a stack of 1650 2D slices. These slices were all converted from 32 to 8 bit and then aligned using the image alignment plugin (TSENG 2011; 2015). This translated each slice of the image to ensure the position of the stem segment remained in the same location across every slice, removing any shifts occurring due to the stem being not mounted perfectly vertically on the CT stage, or not growing perfectly straight.

Next we manually defined a region of the xylem in which embolisms would be analysed (the two yellow circles in (see Figure 3a) for all vertically aligned 2D slices in a single 3D image. The area was selected to maximise the xylem region analysed. The size and shape of this region varied between stem segments due to differences in xylem area and alignment.

To separate embolised fibres from background xylem tissue, and thus allow the area occupied by embolised fibres to be analysed, it was necessary to threshold all slices in the stack. This was done using the local Sauvola thresholding method. We modified the thresholding constant parameter from the default value of 0.5 to 0.2 (decreasing this value increases the region which thresholds to be black).

The choice of thresholding settings allowed us to fully capture embolised fibres, but we also captured embolised vessels (see Figure 3b).

To isolate the fibre embolisms, the individual slices were further processed to remove vessel embolisms without eliminating fibre embolisms. Due to their larger diameter, phase contrast results in vessel embolisms often displaying a much lower signal intensity towards the wall and a higher signal intensity in the centre. This results in embolized vessels being partially resolved. Because vessels did not fully resolve, and because in regions where fibre embolisms are high some fibres embolisms intersect, it was not possible to segment vessels and fibres purely based on size or shape.

The original image stacks were thresholded again, this time using a lower thresholding coefficient to resolve the vessels more clearly. Binary operations (fill holes, erode, dilate) were then used to fill in vessels and separate them from surrounding fibres. The resulting image slices were then able to be segmented based on size and shape to separate vessels and fibres. The vessels identified in these slices were then applied as a mask to remove regions from the original thresholded image (see Figure 3b) containing embolised vessels. Additionally, any embolisms smaller than 6 pixels were removed to avoid any small regions falsely identified as embolisms due to noise present in the images. The postprocessing removed most, but not all embolised vessels from each slice (see Figure 3c). Removing all embolised vessels was not feasible without also removing many fibre embolisms. The full postprocessing algorithm is included as a supplemental file.

# Uncertainty Calculation For Embolised Fibre Fraction

In order to provide an approximate measure of the measurement error the data was analysed for each slice of the image, that is each height. Figure S1 shows the embolised fibre fraction for each slice for one of the trees initially perfused with water (plotted in light red and blue). We show it before and after water perfusion. We plot this in the form of two relative measures, one being the difference between the slice embolised fibre fraction and the total embolised fibre fraction reported in the main text (Figure S1a) and as a relative measure obtained by dividing the slice embolised fibre fraction by the total embolised fibre fraction.

The individual slice results fluctuate due to random variations in thresholding. To capture more general trends reflective of changes in signal or embolism number we use a smoothed dataset (plotted as dark red or blue). This was calculated using the formula:

$$RAT_{i}=\frac{\sum_{i-24}^{i+25} AE_{i}}{\sum_{i-24}^{i+25} AT_{i}}$$

Where the fibre fraction embolised ($RAT$) for layer $i$ is each to the sum of the embolised fibre area ($AE$) of the neighbouring 50 slices divided by the sum of the total xylem area ($AT$) summer for the neighbouring fifty slices. We do this for every slice (from $i=25$ to $i=1710-25$) to get a smoothed distribution that eliminates any minor instantaneous fluctuations in the xylem area embolised.

The error presented in the main text is taken from this smoothed distribution, as it better captures general shifts in response reflecting underlying error. Specifically, the error is based on the maximum and minimum value of the smoothed layer embolised fibre fraction.

It should be noted that this approach is not ideal. Notably, looking at the response of the fresh stem we can see the signal is highest towards the middle and drops off towards the edges, particularly towards the top of the stack. This trend is observed across almost all stems where the embolised fibre fraction is high (i.e. non negligible). It reflects not a true drop in embolised fibre fraction but rather a shift in the signal resulting from variations in the synchrotron beam above and below the centreline. This results in a drop in signal range towards the extremes (particularly the upper limit) which affects thresholding, resulting in the observed trend.


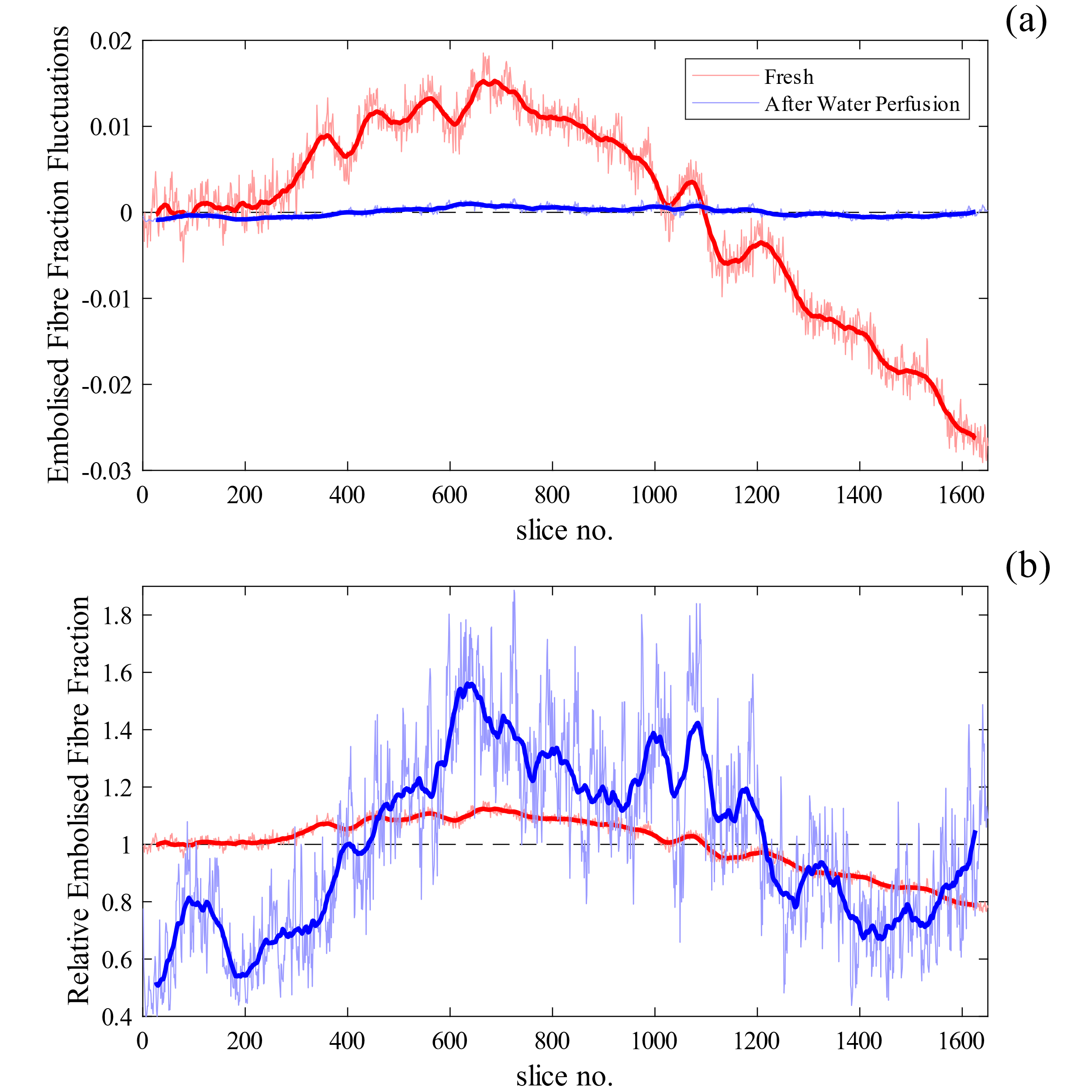


Figure S1. Slice embolised fibre fraction. (a) shows the difference between the slice embolised fibre fraction and the total embolised fibre fraction. (b) shows the ratio of the slice embolised fibre fraction and the average embolised fibre fraction. The dark lines show the smoothed data averaged across 50 slices and the lighter colour the individual slice measurements.

# Statistical Analysis of embolised fibre fraction

In order to further evaluate the changes in the embolised fibre area before and after each treatment we utilise statistical analysis. We perform analysis on the average embolised fibre fraction calculated for the 3D images (as shown in Figure 5 and Figure 7 in the main text). This means that our sample size is very low, which may cause issues with the accuracy of any statistical test. The small sample size also means that there is no way to be certain data is normally distributed (an assumption of the test used).

While we do have data for the embolised fibre fraction in each slice of each 3D image (see Figure S1), the average measure is more appropriate when comparing data from multiple different samples (individual stem segments). Additionally, each slice does not represent an independent sample, being dependent on both behaviour/changes across neighbouring slices and vertical variations in signal associated with the imaging process.

For our experiments we have taken 6 samples (S1-S6) and perfused them first with sucrose solution, then water, then sucrose solution. We have also taken another 6 samples (W1-W6) and perfused them first with water, then sucrose solution, then water. We are interested in evaluating whether there is a statistically significant decrease in the embolised fibre fraction comparing the data before and after each successive perfusion round. To evaluate this we utilise a series of one-tailed paired t-tests comparing a single set of samples (S1-S6 or W1-W6) before and after each perfusion. This analysis was done in Matlab using the ‘ttest’ function (The MathWorks Inc. 2022). We summarise the results of this analysis in Table S1 below. We note the obtained p value, alongside the number of samples taken through that perfusion round ($\leq$6). We also include the change in the mean (across all samples).

For the 4 samples (S1, S2, S7 and S8) taken through the extended perfusion round we did not do a t-test calculation. Because 2 of the samples (S1 and S2) went through 3 prior perfusion rounds, while the other two (S7 and S8) were perfused from fresh, the four samples were not equivalent and could not be grouped to create four samples to evaluate before and after. Evaluating the two groups of two samples separately was possible, but a sample size of 2 was deemed too small for the results of a t-test to have any statistical meaning.

Table S1. Results of one tailed paired t-test analysis comparing the embolised fibre area for each group of samples (sample S1-S6 first perfused with sucrose and samples W1-W6 first perfused with water) before and after each perfusion round. We include the p value, the no. of samples evaluated and the change in the average embolised fibre area across all samples before/after the perfusion.

| Perfusion | p | no. samples | Change in mean |
| --- | --- | --- | --- |
| Samples S1-S6 | | | |
| Sucrose | 0.0718 | 6 | -3.05 |
| Water | 0.0129 | 5 | -6.51 |
| Sucrose | 0.0145 | 5 | -0.86 |
| Samples W1-W6 | | | |
| Water | 0.0049 | 6 | -7.76 |
| Sucrose | 0.8970 | 5 | 0.20 |
| Water | 0.6431 | 4 | 0.02 |

**Embolised Xylem Vessel Behaviour**

The focus of the main text is on the behaviour of fibre embolisms in response to different perfusion treatments. In addition to the embolised fibre fraction, we have also extracted information on the changes in xylem vessel embolisms. Because of the thresholding issues already discussed, in that xylem vessels often don’t threshold fully due to phase contrast, accurately extracting the embolised xylem area is challenging. It would also be useful to extract information on the filled vessels, however the contrast with surrounding tissues was insufficient to segment them.

In order to eliminate xylem embolisms from our analysis of fibre embolisms we used a series of steps to construct an approximate mask which we used to remove xylem embolisms from the thresholded images. While this mask may not fully capture all xylem embolisms, and does distort the shape of some (due to the use of erode/dilate operations), it provides a good approximation for the xylem embolisms present in the sample. We can analyse this mask in the same way as we do fibre embolisms, extracting the xylem area occupied by embolised vessels, computing the overall embolised xylem fraction across all slices, and the uncertainty from the smoothed slice distribution.

The results of this analysis (Figure S2) show that there is little to no clear trend in xylem embolisms. We do see that samples with particularly high embolised vessel fractions (W1, W2 and W5 in Figure S2a and S2 in Figure S2b) show a drop in embolised vessels after their first or second perfusions. This is expected as the positive pressure applied during perfusions should lead to vessel refilling. However, for all other samples the embolised vessel fraction is initially low and any fluctuations are difficult to attribute to a response to perfusion. One sample (4 in Figure S2a) does display an apparent increase in embolised vessels after the final perfusion. However, examining the CT scans, this sample is one with a smaller xylem area on each slice and there is an increase in the no. of vessels in the region scanned for the final scan compared with prior scans, which slightly distorts the results.


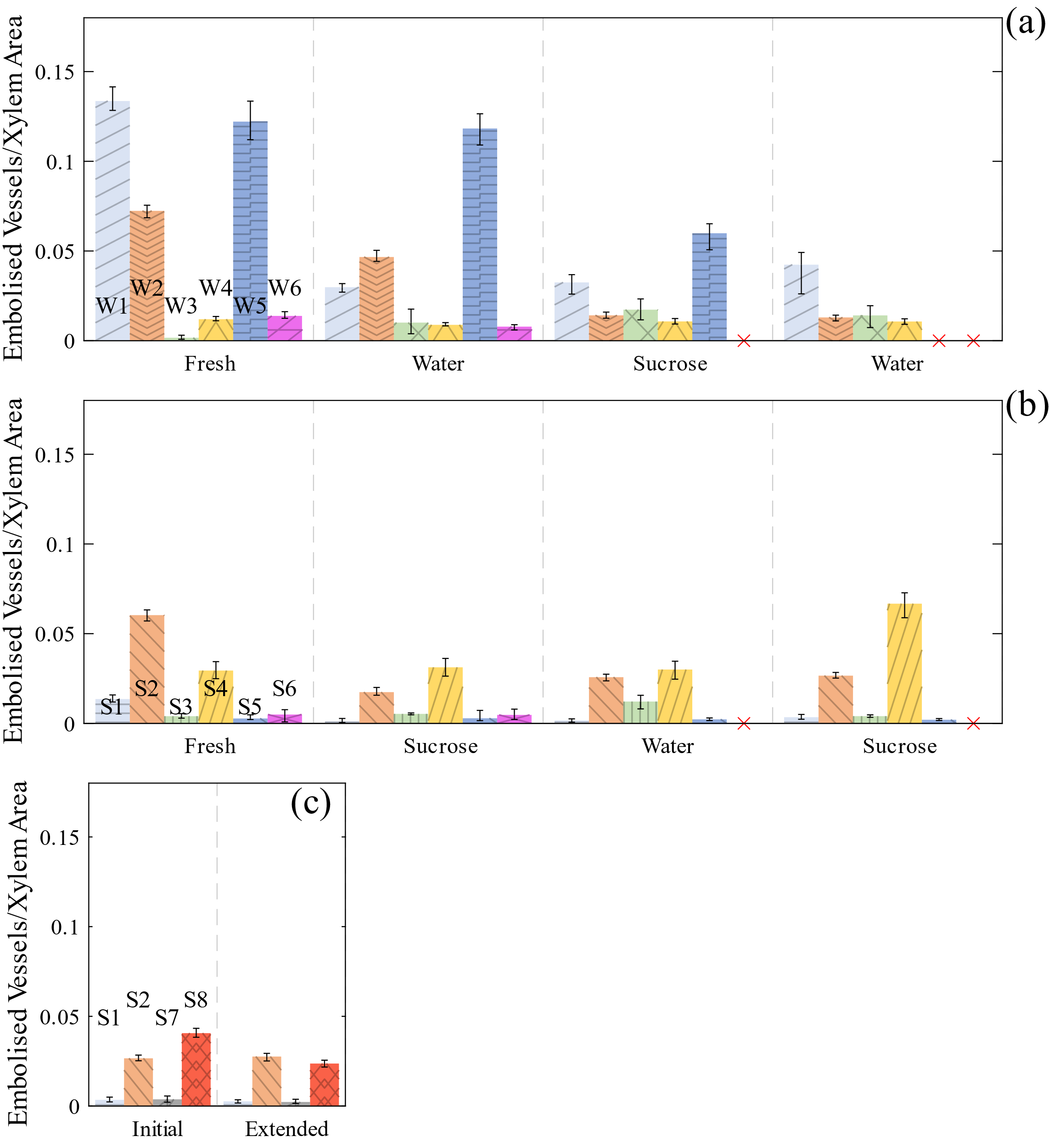


Figure S2: Embolised vessel area as a fraction of the xylem area averaged across all slices. (a) shows the results for samples initially perfused with water. (b) shows the results for samples initially perfused with sucrose solution. (c) shows the results of the extended perfusions of samples S1 and S2 (after their third round of perfusion) and S7 and S8 (perfused from fresh). A red cross through the *x* axis indicates perfusion was not done for that sample. The error bars are the max and min embolised vessel fraction across each slice (1650 in total) of an individual 3D image, after a rolling average was applied to reduce noise.

# Sample Treatments:

The following tables summarise the treatments samples first perfused with water (Table S2) and those first perfused with sucrose (Table S3) were taken through.

Table S2: Noting which perfusion rounds each of the samples first perfused with water were taken through, denoted as yes (Y) *or no (N).*

|  | 2hr Water Perfusion | 2hr Sucrose Perfusion | 2 hr Water Perfusion |
| --- | --- | --- | --- |
| W1 | Y | Y | Y |
| W2 | Y | Y | Y |
| W3 | Y | Y | Y |
| W4 | Y | Y | Y |
| W5 | Y | Y | N |
| W6 | Y | N | N |

Table S3 Noting which perfusion rounds each of the samples first perfused with sucrose were taken through, denoted as yes (Y) or no (N). Samples S7 and S8 were taken through the extended perfusion from fresh, that is they did not undergo any 2 hr perfusions. We have denoted this with N/A.

|  | 2hr Sucrose Perfusion | 2hr Water Perfusion | 2 hr Sucrose Perfusion | Extended Sucrose Perfusion |
| --- | --- | --- | --- | --- |
| S1 | Y | Y | Y | Y |
| S2 | Y | Y | Y | Y |
| S3 | Y | Y | Y | N/A |
| S4 | Y | Y | Y | N/A |
| S5 | Y | Y | Y | N/A |
| S6 | Y | N | N | N/A |
| S7 | N/A | N/A | N/A | Y |
| S8 | N/A | N/A | N/A | Y |

# 3D image Example

Figure S3 is included here to show an example of one of the full 3D images produced using MicroCT.

# Calculation of Tension pressure difference

The pressure difference due to surface tension ($\Delta P_{st}$) is determined via the Young-Laplace equation (Tyree 1995):

$$\Delta P_{st}=\frac{2T}{D}$$

Where $T$ is the surface tension at the air-water interface (0.072 Nm^-1^ from (Tyree 1995)) and $D$ is the bubble diameter. For fibres they can be a range of diameters, with an expected average fibre diameter of $8$µm (Driller et al. 2023). $\Delta P_{st}$ is thus calculated as:

$$\Delta P_{st}=\frac{2\times0.072}{8\times{10}^{-6}}=18000 Pa=18 kPa$$

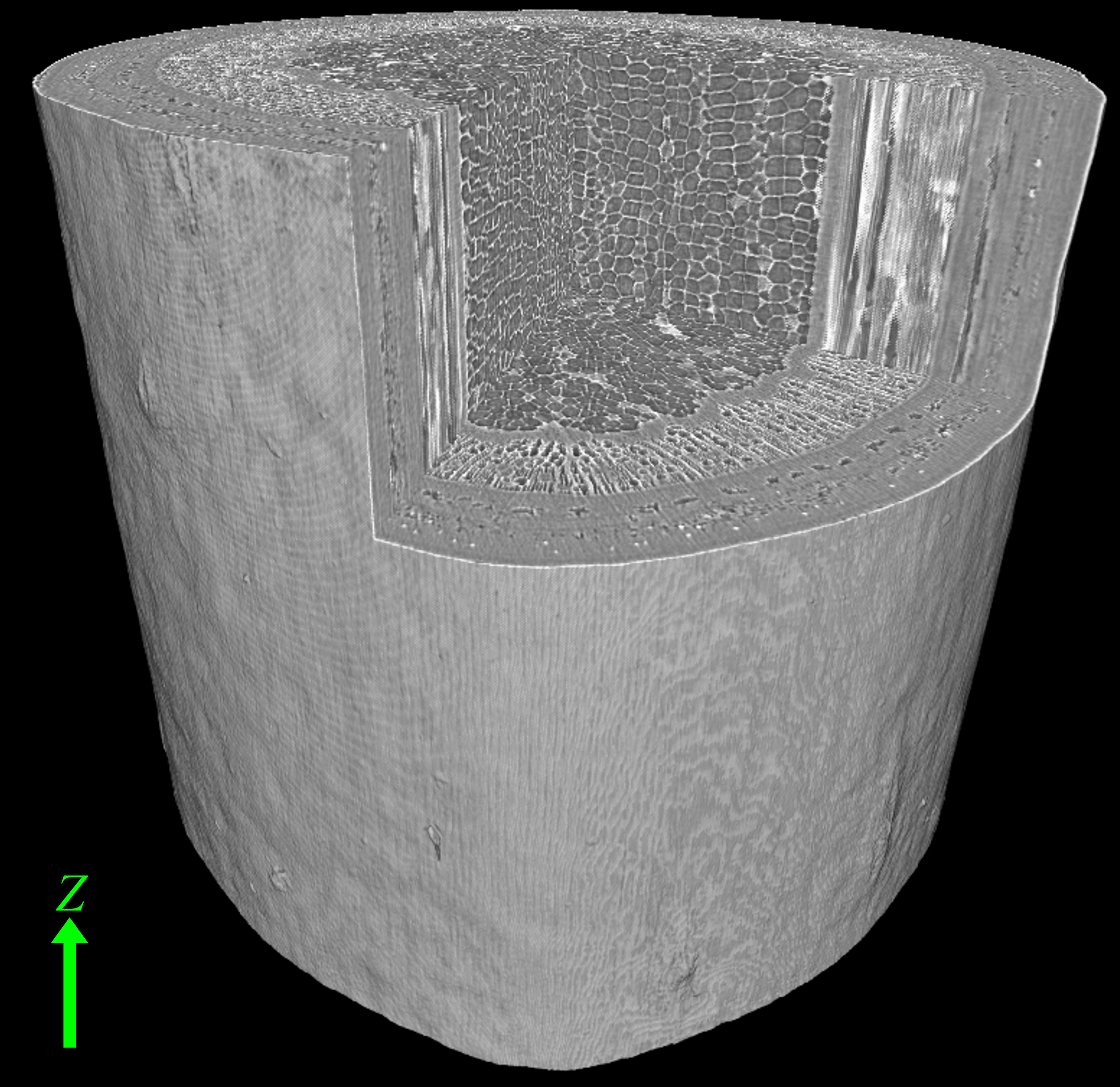


Figure S3: 3D image of 2.64 cm long section of maple stem with background removed. A region has been cut away in order to expose a transverse section perpendicular to the z axis.

# References

Driller T, Robinson JA, Clearwater M, Holland DJ, van den Berg A, Watson M. 2023. Quantitative examination of the anatomy of the juvenile sugar maple xylem. PLOS ONE. 18(10):e0292526.

Schindelin J, Arganda-Carreras I, Frise E, Kaynig V, Longair M, Pietzsch T, Preibisch S, Rueden C, Saalfeld S, Schmid B et al. 2012. Fiji: An open-source platform for biological-image analysis. Nat Methods. 9(7):676-682.

Matlab version: 9.13.0 (r2022b). 2022. Natick, Massachusetts, United States: The MathWorks Inc.; [accessed]. <https://www.mathworks.com>.

TSENG Q. 2011. Study of multicellular architecture with controlled microenvironment. [Grenoble, France]: Université Grenoble.

Template matching and slice alignment--- imagej plugins. 2015. [accessed]. <https://sites.google.com/site/qingzongtseng/template-matching-ij-plugin>.

Tyree M. 1995. The mechanism of maple sap exudation. Paper presented at: 1st International Symposium of Sap Unitilization. Bifuka, Hokkaido, Japan.
